# The Structure and Functions of PRMT5 in Human Diseases

**DOI:** 10.3390/life11101074

**Published:** 2021-10-12

**Authors:** Aishat Motolani, Matthew Martin, Mengyao Sun, Tao Lu

**Affiliations:** 1 Department of Pharmacology & Toxicology, Indiana University School of Medicine, Indianapolis, IN 46202, USA; amotolan@iu.edu (A.M.); mm217@iupui.edu (M.M.); sun19@iu.edu (M.S.); 2 Department of Biochemistry & Molecular Biology, Indiana University School of Medicine, Indianapolis, IN 46202, USA; 3 Department of Medical & Molecular Genetics, Indiana University School of Medicine, Indianapolis, IN 46202, USA; 4 Indiana University Melvin and Bren Simon Comprehensive Cancer Center, Indiana University School of Medicine, Indianapolis, IN 46202, USA

**Keywords:** PRMT5, cancer, cardiovascular disease, neurodegenerative diseases, diabetes, inflammation

## Abstract

Since the discovery of protein arginine methyltransferase 5 (PRMT5) and the resolution of its structure, an increasing number of papers have investigated and delineated the structural and functional role of PRMT5 in diseased conditions. PRMT5 is a type II arginine methyltransferase that catalyzes symmetric dimethylation marks on histones and non-histone proteins. From gene regulation to human development, PRMT5 is involved in many vital biological functions in humans. The role of PRMT5 in various cancers is particularly well-documented, and investigations into the development of better PRMT5 inhibitors to promote tumor regression are ongoing. Notably, emerging studies have demonstrated the pathological contribution of PRMT5 in the progression of inflammatory diseases, such as diabetes, cardiovascular diseases, and neurodegenerative disorders. However, more research in this direction is needed. Herein, we critically review the position of PRMT5 in current literature, including its structure, mechanism of action, regulation, physiological and pathological relevance, and therapeutic strategies.

## 1. Introduction

Arginine methylation is a ubiquitous post-translational modification (PTM) that occurs on both nuclear and cytoplasmic proteins [1]. It is catalyzed by a family of enzymes named protein arginine methyltransferases (PRMTs) [1,2]. So far, nine PRMTs have been identified in human cells: PRMT1, 2, 3, 4 (also known as co-activator-associated arginine methyltransferase 1, CARM1), 5, 6, 7, 8, and 9 (also known as F-box only protein 11, FBXO11) [3].

Depending on the type of methylarginine that is introduced, PRMTs are classified into four types: type I, II, III, and IV. Type I, II, and III PRMTs transfer methyl groups from S-adenosylmethionine (SAM) to a terminal ω-guanidine nitrogen of protein arginine residue, generating methylarginine and another, product S-adenosylhomocysteine (SAH) [4]. Whereas a type IV PRMT methylates the internal (or δ) guanidino nitrogen, generating monomethylarginine, which has been only described in yeast [3,5]. In a stepwise manner, type I and type II PRMTs first methylate arginine residues to result in ω-*NG*-mono- methylarginine (MMA), which acts as an intermediate. Subsequently, type I PRMTs (PRMT1, 2, 3, 4, 6, and 8) catalyze the formation of asymmetric ω-*NG, NG*-dimethylarginine (ADMA), while type II PRMTs (PRMT5, PRMT7, and PRMT9) catalyze the formation of symmetric ω-*NG, N’G*-dimethylarginine (SDMA) [6]. PRMT7 also exhibits type III PRMT activity by catalyzing the monomethylation of certain substrates without further formation of SDMA [3,7,8] (Table 1). Notably, with an assigned designation from the HUGO Gene Nomenclature Committee, PRMT10 is now also referred to as PRMT9.

PRMTs are generally expressed in tissues and can methylate both histone and non-histone proteins. Methylation of arginine residues is critical to various biological processes, including cellular signaling transduction, mRNA splicing, transcription, DNA damage repair, cell proliferation and differentiation, and protein-protein interactions [9,10,11,12]. Notably, the deregulation of PRMT enzymes is implicated in the pathogenesis of different diseases, including cancer, metabolic diseases, neurodegenerative diseases, cardiovascular diseases, aging, and so on [13]. Particularly, the role of arginine methylation has been extensively researched in various human diseases, such as cancer, diabetes, Alzheimer’s disease, and cardiovascular disease [14]. Further insights into the distinct roles of the different PRMTs are anticipated to provide new approaches to disease prevention, diagnosis, and treatment [15]. Among the PRMT family members, PRMT5 has become increasingly attractive as a therapeutic target for small molecule inhibition [16,17]. This review discusses PRMT5’s structure and function, including its role in human diseases and promising therapeutic treatments.

## 2. Structure and General Function of PRMT5

### 2.1. Human PRMT5 Structure and Mechanism of Action

**Figure 1:**
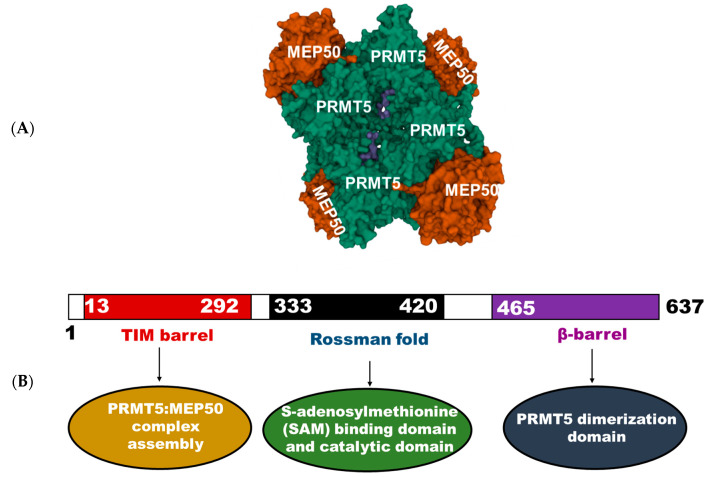
(**A**) Crystal structure of human PRMT5 in a heterooctameric complex with MEP50 (PDB ID: 4GQB). (**B**) Structural and functional domains of PRMT5.

Approximately 20 years ago, Pollack and colleagues studied the function of Janus kinase 2 (JAK2) in cell signaling, wherein they aimed to identify proteins that interact with JAK2. In this study, PRMT5, then known as Jak-binding protein 1 (JBP), was discovered and shown to possess methyltransferase activity [18]. Ten years later, the first crystal structure of human PRMT5 was resolved and described by Antonysamy et al. [19]. The human PRMT5, which is 637 amino acids long, commonly associates with methylosome protein 50 (MEP50) in a 435 kDa heterooctameric complex. MEP50 is a tryptophan-aspartic acid (WD) repeat-containing protein and acts to stabilize the PRMT5 complex and potentiate its methyltransferase activity. Thus, PRMT5 oligomerizes to form an inner core tetramer, with four surrounding MEP50 molecules bound to the N-terminal of PRMT5 (Figure 1). This unique PRMT5:MEP50 complex further interacts with several partner proteins—such as B lymphocyte-induced maturation protein (Blimp1), RIO kinase 1 (RioK1), menin, pICLn, methyl-CpG-binding domain/nucleosome remodeling deacetylase (MBD/NuRD), and coordinator of PRMT5 (COPR5)—in a context-dependent manner to enable a diverse range of substrate specificities and biological functions [19]. As shown in Figure 1, the structural composition of PRMT5 includes an N-terminal TIM barrel domain, which aids the assembly of the PRMT5:MEP50 complex. This is followed by a middle Rossman fold domain that is responsible for binding SAM and catalysis. The Rossman fold domain also has a general methyltransferase structure that facilitates the catalytic activity of PRMT5 [19]. Then, a C-terminal β-barrel domain aids dimerization of PRMT5 to form the inner core tetramer in the PRMT5:MEP50 complex [20].

**Figure 2:**
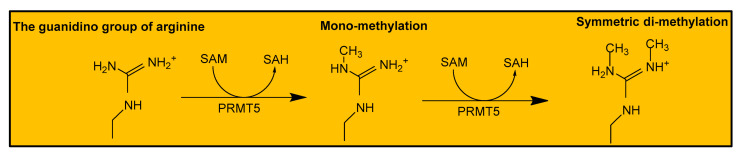
Scheme of the generation of dimethylarginine by PRMT5.

As already mentioned, PRMT5 is a type II PRMT that symmetrically mono- or di-methylates its substrates by catalyzing the transfer of methyl groups from SAM to arginine residues on a substrate protein (Figure 2). This results in the formation of mono- or di-methylarginine and one or two molecules of SAH [21]. The methyltransferase function of PRMT5 is facilitated by critical conserved residues within the Rossman fold and the β-barrel domain. For example, during the methylation of a substrate protein such as histone 4 (H4), the arginine residue of H4 binds into a tunnel-like region composed of tryptophan (W) 579, phenylalanine (F) 327, and leucine (L) 312. These residues form favorable interactions with the substrate protein, thus enabling the arginine residue access to PRMT5’s active site. The active site of PRMT5 contains two conserved glutamate (E) residues, E435 and E444, which form two hydrogen bond with the terminal guanidino nitrogen atom of arginine [9]. Then, F327 further orients the substrate’s arginine for efficient transfer of the methyl group. F327 has been shown to reinforce the specificity of PRMT5 for the generation of symmetric dimethylation products. The product specificity role of F327 is evident from the formation of asymmetric dimethylated H4R3 when F327 is mutated to methionine [20]. Collectively, these critical residues of PRMT5 position a substrate protein in the best conformation for efficient catalysis, leading to the modulation of a protein’s function.

Similarly, with regard to sequence specificity, PRMT5 has an increased preference to methylate arginine residues that are sandwiched between two glycine residues (GRG motif) on RNA processing proteins [22]. A separate study with *C. elegans* PRMT5 (cPRMT5), which has 34% sequence similarity with human PRMT5, showed that the presence of positively charged residues downstream of arginine is essential for high-affinity binding to a substrate [23]. These studies indicate that PRMT5 targets specific arginine residues based on the surrounding sequences of a substrate.

### 2.2. Cellular Function of PRMT5

The activity of PRMT5 is essential to a wide range of biological processes, including cellular growth and development, differentiation, chromatin regulation, splicing, translation, DNA damage response, protein trafficking, and cell signaling [24]. PRMT5 is responsible for the methylation of various proteins like histones H2A, H3, and H4, transcription factors, cell receptors, etc., to regulate their physiological functions [24]. It has been well-established that the symmetric dimethylation of arginine 3 of H2A (H2AR3me2s) and H4 (H4R3me2s) suppresses gene transcription [25]. These repressive methylation marks exert their biological effects on different systems and cellular processes. For example, in conjunction with MEP50, PRMT5 maintains embryonic stem cell (ESC) pluripotency via dimethylation of H2AR3, leading to the downregulation of several differentiation genes such as *GATA4*, *6*, and *HOXD9*. The knock-out of the *PRMT5* gene in mice also causes early embryonic lethality as it prevents the pluripotency of blastocysts [25]. Because of its extensive role in embryonic development and differentiation, PRMT5 has been termed the guardian of the germline [26]. In addition, PRMT5 plays a role in immune function. A recent study demonstrated that PRMT5 is essential for T-cell activation and proliferation and necessary for activated B-cell survival, maturation, proliferation, and antibody production [27,28]. PRMT5 is also expressed in the nervous system tissues, and it promotes the development and differentiation of oligodendrocyte progenitor cells—a major cell type responsible for myelin production, via deposition of methylation marks on H4R3 and inhibition of lysine acetylation on H4 [29].

In addition to histone methylation and its role in cellular development, PRMT5 coordinates other cellular processes through the methylation of diverse proteins. For example, PRMT5 activity is critical to hematopoiesis. It helps to maintain the viability and functions of hematopoietic stem cells (HSCs) through the repression of tumor protein p53 (p53), mechanistic target of rapamycin kinase (mTOR) signaling, and via regulation of the splicing of DNA repair genes [30]. Similarly, PRMT5 methylates several transcription factors, such as p53, E2F transcription factor 1 (E2F-1), and nuclear factor kappa B (NF-κB), to regulate cellular apoptosis, cell cycle progression, and inflammation [24,31]. The growing evidence of PRMT5 function on different physiological processes demonstrates its potential as a viable drug target in diseases.

## 3. Regulation of PRMT5

Since PRMT5 is highly involved in orchestrating several critical biological processes, its cellular function needs to be tightly regulated. Multiple sources of evidence in the literature have shown that the PRMT5’s activity is positively or negatively regulated via different mechanisms, including the alteration of its subcellular localization, turnover rate, and substrate specificity [32]. These distinct mechanisms can be mediated by PTMs, microRNAs (miRNA), and/or interaction with partner proteins (Table 2).

### 3.1. Regulation by PTMs

PTM involves the addition or removal of distinct chemical groups on proteins to broaden proteins’ functions [14,33]. To date, two main PTMs have been reported to modulate PRMT5 function: methylation and phosphorylation. For instance, the asymmetric methylation of PRMT5 by coactivator-associated arginine methyltransferase 1 (CARM1) at the evolutionary conserved arginine(R) 505 is essential for PRMT5 homodimerization, and the abrogation of this R505 methylation results in impaired methyltransferase activity [34]. Similarly, there are several lines of evidence documenting the regulatory role of phosphorylation on PRMT5’s cellular activity. For example, Espejo and colleagues reported that the phosphorylation of PRMT5 by Akt and serum- and glucocorticoid-inducible kinases (SGK) at threonine (T)634 regulates PRMT5 subcellular localization and promotes its interaction with proteins containing 14-3-3 motifs rather than those containing PDZ motifs [35]. This switch in PRMT5 protein interaction may result in the sequestration of PRMT5 or aid the methylation of a specific set of substrates. Also, two tyrosine(Y) residues on PRMT5, Y304 and 307, known to be phosphorylated by JAK2, facilitate the substrate protein-binding for efficient catalysis [19]. Notably, our laboratory recently uncovered another key PRMT5 residue modified by phosphorylation. In this study, we discovered that PRMT5 is phosphorylated on serine (S)15 by protein kinase C iota (PKCι). This S15 phosphorylation was shown to be critical for NF-κB activation and to regulate the expression of a subgroup of NF-κB target genes in HEK 293 cells [36]. Additionally, in breast cancer cells, PRMT5 is phosphorylated at T139 and 144 by LKB1, and the mutation of these sites leads to a significant decrease in PRMT5 catalytic activity and reduces the interaction with its co-factors—MEP50, pICln, and RiOK1 [37]. Although less characterized, the ubiquitination of PRMT5 by carboxyl terminus of heat shock cognate 70-interacting protein (CHIP) at multiple lysine residues has been reported to cause PRMT5 proteasomal degradation in prostate cancer cells [38]. Taken together, these studies document the diverse regulatory impact of PTMs on the functions of PRMT5 in cells.

### 3.2. Regulation by miRNA

PRMT5 modulates the expression of several miRNA via its methyltransferase activity on histones at miRNA promoters [39]. Conversely, miRNA can also regulate PRMT5 expression through distinct mechanisms. In a previous study, Pal and colleagues showed that low PRMT5 levels in normal B cells were maintained by significantly higher expression levels of miR-92b and miR-96, both of which are low in transformed B-cells. miR-92b and miR-96 inhibit PRMT5 translation by binding to the 3′UTR of PRMT5 mRNA, and this inhibition, in turn, suppresses the expression of the PRMT5 target gene—the suppressor of tumorigenicity 7 (ST7) [40]. The same group also reported that miR-19a, miR-25, and miR-32 inhibit PRMT5 expression in transformed B cells compared to normal B cells by a similar mechanism [41]. These studies underscore the importance of miRNA in maintaining the normal function and turnover of PRMT5, and how miRNA dysregulation can lead to aberrant PRMT5 expression.

### 3.3. Regulation by Interacting Proteins

The substrate specificity and cellular function of PRMT5 are often directed by its associating, binding partner proteins [32]. For example, PRMT5, when bound to the cooperator of PRMT5 (COPR5), preferentially methylates H4R3 rather than H3R8. Thus, COPR5 serves as an important adaptor for the recruitment of PRMT5 to the chromatin [42]. Also, PRMT5 interacts with the human SWItch/Sucrose Non-Fermentable (hSWI/SNF) chromatin, remodeling enzymes to methylate H3R8 and H4R3 at the promoters of ST7 and the nonmetastatic 23 (NM23) gene, to regulate cell growth [43]. Another chromatin remodeling complex, MBD2/NuRD, associates with the PRMT5:MEP50 complex and recruits the complex to the CpG islands of p14^ARF^ and p16^INK4a^, thereby suggesting a role of MBD2/NuRD in regulating PRMT5 repressive activity on the endogenous inhibitors of the cell cycle [44]. Similarly, a scaffold protein, menin, is known to bind to PRMT5 and recruit it to the promoter of growth arrest specific 1 (Gas1) gene as a corepressor, to antagonize Sonic Hedgehog signaling in pancreatic islets [45]. Also, Blimp1 binds to PRMT5 in primordial germ cells to promote the symmetric dimethylation of H2AR3 and H4R3, and Blimp1 directs PRMT5-mediated repression of a subset of genes involved in the cell cycle, cell signaling, metabolism, and transcription [46]. In HeLa cells, Ski proteins were found to associate with PRMT5, alongside histone deacetylase 3 (HDAC3) and mothers against decapentaplegic homolog 2/3/4 (Smad2/3/4) proteins, to maintain the transcriptionally repressive state of Smad7 in the absence of TGF-β [47]. On the other hand, PRMT5 functions as a co-activator in its cooperation with pICln via the symmetric dimethylation of H4R3 at the promoter of genes involved in DNA double-stranded break repair [48]. Also, the association of PRMT5 with pICln and WDR77 promotes the recruitment of spliceosomal proteins, such as SmD1 and SmD3, to PRMT5 for methylation. This event is integral to the assembly of pre-mRNA splicing machinery [49]. Notably, RioK1 and pICln bind competitively to the PRMT5:MEP50 complex, with RioK1 interaction, promoting the recruitment of nucleolin to the complex for methylation [50]. Nucleolin is essential to ribosomal maturation and synthesis [50]. Thus, the aforementioned studies suggest that PRMT5’s role in transcription, RNA processing, or translation can be modulated by specific proteins interacting with the PRMT5:MEP50 complex (Table 2).

## 4. Role of PRMT5 in Human Diseases

### 4.1. PRMT5 in Cancer

A growing number of studies have established the role of PRMT5 as a tumor promoting factor in several types of cancers. Owing to its methyltransferase activity on the histones and oncoproteins, PRMT5’s role in cancers is entrenched in distinct cellular processes like cell signaling, DNA damage response, gene regulation, and splicing, among others [51]. Particularly, dysregulation of PRMT5 is critical for the progression of hematologic malignancies. For example, in lymphoma cell lines, PRMT5 is upregulated and increases the expression of pro-survival proteins like cyclin D1, c-myc, and survivin. This occurs via the deposition of repressive methylation marks on H3R8 in the promoter region of *AXIN2* and *WIF1*, both of which are negative regulators of wnt/β-catenin signaling [52]. Similarly, in vivo studies showed that the tumorigenesis in lymphocytes driven by oncogenes such as cyclin D1 requires high PRMT5 expression, and that this increased PRMT5 expression further antagonizes the apoptotic function of p53 via arginine methylation [53]. In mantle cell lymphoma (MCL), low levels of miR-92b and miR-96 drive increased PRMT5 expression, promoting cell proliferation [40]. Another study reported that PRMT5 interaction with tripartite motif-containing protein 21 (TRIM21), an IKKβ ubiquitin ligase, inhibits IKKβ degradation in multiple myeloma, thereby inducing NF-κB signaling and cell growth of multiple myeloma cells [54]. Taken together, convincing literature documents the pertinent role of aberrant PRMT5 expression in promoting major cancer hallmarks in hematologic cancers.

In addition, PRMT5 promotes oncogenicity in various solid cancers, including colon, breast, prostate, lung, liver, bone, skin, ovarian, gastric, brain, and pancreatic cancers, among others [55]. For instance, PRMT5 aids cellular proliferation, migration, and invasion in breast cancer cells by inhibiting the expression of Dickkopf WNT signaling pathway inhibitor 1 (DKK1) and DKK3, known antagonists of the wnt/β-catenin pathway [56]. In hepatocellular carcinoma, PRMT5-catalyzed repressive dimethylation on H4R3 at the B-cell translocation gene 2 (BTG2) promoter increases cell proliferation through the ERK signaling pathway [57]. Similarly, in lung cancer, PRMT5 induces the downregulation of tumor suppressor genes, such as GLI pathogenesis related 1 (GLIPR1), leprecan-like 1 (Leprel1), and BTG2, and the upregulation of growth factors such as fibroblast growth factor receptor substrate 1/2/3/4 (FGFR1/2/3/4) and human epidermal growth factor receptor 2/3 (HER2/3), thereby enhancing cell growth [58]. Particularly, PRMT5-mediated increased FGFR3 signaling is caused by silencing of the miR-99 family, which negatively regulates the expression of FGFR3 in lung cancer [59]. Interestingly, our laboratory reported that PRMT5-catalyzed dimethylation on R30 of the NF-κB p65 subunit and R205 of YBX1 promotes cell proliferation, migration, and anchorage-independent growth in CRC, suggesting the versatility of a PRMT5-regulated substrate in CRC tumors [31,60]. We further showed that PRMT5 inhibition significantly decreases the survival of colorectal and pancreatic cancer cell lines [17]. From a clinical perspective, high PRMT5 has been associated with a poor prognosis in patients with breast cancer, hepatocellular carcinoma, lung cancer, ovarian, and gastric cancer [61,62,63]. Notably, PRMT5’s high nuclear expression has been suggested as a potential biomarker for assessing submucosal invasion of tumors resected in the early stage of CRC. A similar prognostic potential of high PRMT5 expression in the nucleus and/or the cytoplasm has been reported in brain, lung, ovarian, skin, and prostate cancers [64]. Collectively, these lines of evidence demonstrate the extensive oncogenic role of PRMT5 in cancers and its potential value as a clinical biomarker to improve patients’ treatment modalities.

### 4.2. PRMT5 in Diabetes

Beyond cancer, evidence suggests that PRMT5 plays an important role in diabetes. Type 2 diabetes mellitus (T2DM), which accounts for over 90% of diabetic patients, is mainly characterized by the dysfunction of pancreatic β-cells, resulting in defective insulin release and insulin resistance [65]. Other associating pathophysiologies of T2DM include hyperglycemia, hyperlipidemia, mitochondrial dysfunction, inflammation, and increased reactive oxygen species (ROS) levels [66]. Interestingly, PRMT5 has been reported to play a role in metabolic pathways that perpetuate the pathologies of T2DM. For instance, in white adipose tissue, PRMT5 methylates sterol regulatory element-binding transcription factor 1a (SREBP1a) to enhance triacylglycerol formation [67]. PRMT5 also methylates the transcription elongation factor SPT5 to promote lipid droplet biogenesis [67]. A separate study also reported that PRMT5 serves as a coactivator for the expression of adipogenic genes, such as peroxisome proliferator-activated receptor γ2 (PPARγ2), adipocyte protein 2 (aP2), adiponectin, leptin, and resistin [68]. This demonstrates the regulatory role of PRMT5 in the metabolism of fatty acids and thus insulin sensitivity. Similarly, in association with the menin scaffold protein, PRMT5 reduces the expression of glucagon-like-peptide-1 (GLP1) and dimethylates cAMP responsive element binding protein (CREB) and forkhead box O1 (FOXO1) to block protein kinase A (PKA)-mediated phosphorylation [69]. Another group showed that, in response to glucagon, PRMT5 promotes phosphorylation of CREB via PRMT5 binding to the CREB regulated transcription coactivator 2 (CRTC2) promoter, leading to increased expression of gluconeogenic genes. Notably, the increased activity of CREB and CRTC2 is also observed in diabetes and contributes to hyperglycemia [70]. In summary, these events orchestrated by PRMT5 play a key role in suppressing pancreatic β-cell function and in regulating glucose homeostasis. On the contrary, a study conducted by Ma and colleagues reported that the conditional knockout of PRMT5 in islet cells of the pancreas caused defects in glucose tolerance and glucose-stimulated insulin release in β-cells [71]. The proposed mechanism suggests that PRMT5 dimethylates H3R8 to increase the binding of the brahma-related gene-1 (BRG1) chromatin remodeling enzyme to the insulin promoter, to increase insulin production. This unusual observation was attributed to a compensatory mechanism of elevated β-cell proliferation induced by impaired insulin production on the PRMT5 knockout mice [71]. Thus, further studies are required to understand the nuanced role of PRMT5 in T2DM models, to aid better exploration of PRMT5 as a viable therapeutic target in diabetes.

### 4.3. PRMT5 in Cardiovascular Diseases

Cardiovascular disease is a category of disease that occurs in the heart or blood vessels [72]. The number of studies investigating the role of PRMT5 in cardiovascular diseases is limited. However, reports published recently suggest that differential PRMT5 levels may serve as a risk indicator for developing certain cardiovascular diseases or may reduce/promote its related morbidities. In cellular models of cardiomyocyte hypertrophy, a form of heart enlargement that causes heart failure, overexpression of PRMT5 results in the reduction of isoprenaline-induced hypertrophy through repressive methylation of *HOXA9*, a gene that plays a critical role in the development of several cardiovascular diseases [73]. This finding corroborates a previous report that demonstrates the role of PRMT5 in suppressing the expression of hypertrophic genes in cardiomyocytes via methylation of *GATA4*, a transcription factor that regulates cardiac remodeling [74]. Notably, in the peripheral blood obtained from 178 patients with acute myocardial infarction (AMI) and stable coronary artery disease (CAD), PRMT5 was significantly lower in AMI patients compared to stable CAD patients [75]. Thus, this study suggests that low PRMT5 expression in the blood may serve as a biomarker for individuals with increased risk for AMI development. In contrast, increased PRMT5 expression may enhance the pathological progression of inflammatory-driven cardiovascular diseases. For example, the high expression of C-X-C motif chemokine ligand 10 (CXCL10), a chemokine that extensively contributes to atherosclerosis and coronary artery disease in endothelial cells, is driven in part by PRMT5 methylation of NF-κB at R30 and R35. This event was observed in response to tumor necrosis factor-alpha (TNF-α), a potent activator of NF-κB signaling [76]. A follow-up study by the same group reported that, in response to TNF-α and interferon gamma (IFN-γ), PRMT5-induced methylation of p65 at R174 increases the expression of CXCL11, another chemokine that worsens the atherosclerosis pathology [77]. Collectively, these discussed studies suggest that the role of PRMT5 is context-dependent, and therefore, the elucidation of the PRMT5 molecular activity in contribution to different cardiovascular diseases is an area worthy of further exploration.

### 4.4. PRMT5 in Neurodegenerative Diseases

The role of PRMT5 in neurodegenerative diseases is not well studied. Given that a high expression of PRMT5 has been identified in the human brain, a few studies delineated the role of PRMT5 in neurodegenerative disorders [78]. For instance, Alzheimer’s disease (AD) is one of the most common neurodegenerative diseases. It is often characterized by the accumulation of amyloid-β production, which induces neuronal death [79]. Interestingly, in a human AD cell model, the depletion of PRMT5 was reported to induce cell death and trigger apoptosis in neurons when indued by Aβ [80]. It is worthwhile to note that in microglial cells, activation of NF-κB signaling exacerbates the AD pathology via upregulation of cytokines that aid neuroinflammation and the formation of Aβ plaques [81,82]. However, the link between PRMT5 and NF-κB is yet to be examined in glial cells. In a human neuroblastoma cell line with overexpression of Swedish mutant of human amyloid-β precursor protein, PRMT5 overexpression resulted in reduced expression of E2F-1, p53, and Bax, and increased levels of glycogen synthase kinase 3β (GSK-3β), all of which prevent apoptosis [80].

In addition, the importance of PRMT5 in preventing Huntington’s disease (HD) has been suggested. HD is an autosomal dominant neurogenerative disease, the cause of which is often attributed to the presence of mutant polyglutamine sequence in the huntingtin (Htt) protein [83]. According to a study by Ratovitski and colleagues, the methyltransferase activity of PRMT5 on histones is severely impaired by the mutant Htt protein, and the ectopic expression of PRMT5 enhances the survival of neuronal cells expressing mutant Htt [84]. In conclusion, in several studies, PRMT5 has been suggested to play a protective role in terms of neuronal cells. However, studies to examine its role in other brain cell types, such as microglia and astrocytes, within the context of neurodegeneration, are missing. This research gap certainly warrants future clarification of the overall role of PRMT5 in human neurodegenerative diseases. As the brain has several important cell types, the overall effect of PRMT5 in the human brain is determined by its integrated role in all the cell types, instead of in just a couple of them.

## 5. Targeting PRMT5 in Human Diseases

Because PRMT5 is a critical regulator of several systems, it is unsurprising that the overexpression or dysregulation of PRMT5 is associated with several disease states, including several hematologic and solid-state cancers [61,85,86]. Furthermore, the dysregulation and upregulation of PRMT5 in several cardiovascular disorders [75] and neurological disorders [80,84] raise the question of how PRMT5 may be therapeutically targeted for disease treatment. The breakdown of potential therapeutics for PRMT5 can be broadly classified into targeted and non-specific. These therapeutics are shown in Table 3. Several small molecule inhibitors of PRMT5 have been developed and are currently going into or through clinical trials. Many of these small molecules act by blocking the binding of SAM to PRMT5, either through competitive or non-competitive binding. In some cases, the mechanism of inhibition is unknown. However, most of the inhibitors are known to work through direct binding to the catalytic region of PRMT5 (direct inhibition). The GSK inhibitor (EPZ015938/GSK3326595) was the first PRMT5 inhibitor to be studied in a clinical setting, but there were mixed results of the Meteor-1 Phase I trial. Adenoid cystic carcinoma (ACC) was the primary indication for use of this compound. High dosages were required (400 mg, QD) to see any benefit, and adverse events were observed at multiple dosing levels, including the 400 mg dose (Table 3). Currently, this GSK inhibitor, EPZ015938/GSK3326595, is in phase I/II trials for leukemias as well as solid state cancers [87]. Furthermore, EPZ015938/GSK3326595 is on course for planned clinical trials relating to breast cancer and solid-state cancers (NCT02783300, NCT04676516). Another example is GSK3186000A, which was used in leukemia cells to reduce PRMT5 activity and may represent a potentially useful future therapy [88]. JNJ-64619178 is a small molecule inhibitor used in phase I clinical trials in brain cancers and advanced solid-state tumors [89]. Clinical trials are also underway for a new PRMT5 small molecule inhibitor PF-06939999 developed to treat esophageal cancers as well as small lung cell carcinoma [90]. Other therapeutics have been developed to directly target the SAM binding pocket by larger pharmaceutical companies but have not yet gone into clinical trials. These include LLY-283 by Eli Lilly and company, which has been shown to reduce tumor growth in skin cancer [91]. CMP-5 is a compound developed by Merck et al. that has been used to block SAM binding in glioblastoma [92,93]. MRTX9768, a compound developed by Miratis Therapeutics, Inc., is focused on inhibiting the methylthioadenosine phosphorylase (MTA)-PRMT5 complex in MTAP^del^ cancer cells, resulting in their targeting for destruction [94]. PRT543 and PRT811, developed by Prelude Therapeutics, are further inhibitors that directly bind to the SAM binding pocket; these are slated for clinical trials soon (NCT03886831 and NCT04089449) [95]. PR5-LL-CM01, developed by our lab and further licensed to EQon Pharmaceuticals, shows great efficacy in tumor inhibition in pancreatic cancer, colon cancer, and breast cancer [17]. Furthermore, the use of most PRMT5 inhibitors is almost exclusively focused on the treatment of cancers, and in almost all cases, the mode of inhibition is direct. This opens up a wide range of therapeutic applications for PRMT5 inhibitors in other diseases such as cardiovascular disease, and perhaps neurodegenerative disorders, etc.

## 6. Perspective and Conclusions

The recent explosion of PRMT5 research in various disease model systems is indicative of its enormous potential as a drug target to inhibit the progression of human diseases. Undoubtedly, PRMT5 is an important type II arginine methyltransferase with diverse substrates and functions in humans. PRMT5’s unique heterooctameric structure facilitates its interaction with co-factors, partner proteins, and substrates, thus helping to maintain the genomic integrity, signal transduction, and development of cells. The clinical importance of PRMT5 is evident from its extensive role in driving or reducing inflammatory diseases, such as cancer, diabetes, neurodegeneration, and cardiovascular disease. Thus, the current knowledge on PRMT5 in the literature can be translated to improve the outcomes of patients with related diseases. Notably, our group was one of the first to establish the link of PRMT5 to inflammation through its methyltransferase activity on the NF-κB signaling pathway. Together with other groups, we have demonstrated the pathological relevance of the PRMT5/NF-κB axis in pancreatic cancer, colon cancer, and heart disease [31,76]. However, more studies are needed to buttress the role of the PRMT5/NF-κB signaling axis in AD, considering that the activation of NF-κB in surrounding glia cells contributes to neuroinflammation [81]. Hence, future work studying PRMT5 should investigate how the differential regulation of PRMT5 leads to its tissue-dependent function, and vice versa, thus defining under what conditions PRMT5 plays a role in diabetes and cardiovascular disease, as well as examining strategies to overcome the existential barriers to effective PRMT5 targeted therapies in human diseases.

## Figures and Tables

**Table 1:** Human PRMTs superfamily.

Member	Type	Methylation Pattern	References
PRMT1	I	Monomethylation and asymmetric dimethylation	[3]
PRMT2	I	Monomethylation and asymmetric dimethylation	[3]
PRMT3	I	Monomethylation and asymmetric dimethylation	[3]
PRMT4	I	Monomethylation and asymmetric dimethylation	[3]
PRMT5	II	Monomethylation and symmetric dimethylation	[3]
PRMT6	I	Monomethylation and asymmetric dimethylation	[3]
PRMT7	II and III	Monomethylation OR symmetric dimethylation	[3]
PRMT8	I	Monomethylation and asymmetric dimethylation	[3]
PRMT9/FBXO11/PRMT10	II	Monomethylation and symmetric dimethylation	[3]

**Table 2:** Summary of the distinct mechanisms of PRMT5 regulation.

Regulation	Regulators	Mechanism	Effect	Reference
PTMs	Coactivator-associated arginine methyltransferase 1 (CARM1)	Methylation of R505	Promotes PRMT5 homodimerization	[34]
	Protein kinase B/Akt	Phosphorylation of T634	Aids interaction with 14-3-3-proteins	[35]
	Janus kinase 2 (JAK2)	Phosphorylation of Y304 and Y307	Increases substrate binding	
	Protein Kinase C iota (PKCι)	Phosphorylation of S15	Promotes NF-κB activation	[36]
	Liver kinase B1 (LKB1)	Phosphorylation of T139 and T144	Increases methyltransferase activity and interaction with co-factors	[37]
miRNAs	miR-19a, miR-25, miR-32 miR-92b, and miR-96	Binds to 3′UTR of PRMT5 mRNA	Reduces PRMT5 levels	[40]
Protein Interactions	Coordinator Of PRMT5 (COPR5)	Serves as an adaptor for PRMT5 recruitment to chromatin	Causes PRMT5 preferential methylation of H4R3	[42]
	Human SWItch/Sucrose Non-Fermentable (hSWI/SNF)	Recruits PRMT5 to H3R8 and H4R3 at ST7 and NM23 promoters	Reduces expression of ST7 and NM23	[43]
	Methyl-CpG-binding domain protein 2/nucleosome remodeling and deacetylase (MBD2/NuRD)	Recruits PRMT5 to CpG islands of p14^ARF^ and p16^INK4a^	Reduces expression of p14^ARF^ and p16^INK4a^	[44]
	Menin	Recruits PRMT5 to Gas1 promoter	Reduces Gas1 gene expression and enhances Sonic Hedgehog signaling	[45]
	B-Lymphocyte induced maturation protein-1 (Blimp1)	Recruits PRMT5 to H2AR3 and H4R3	Repression of genes in cell cycle, cell signaling, metabolism and transcription	[46]
	pICln	Recruits PRMT5 to H4R3 and spliceosomal proteins	Repression of genes in DNA double-stranded break; Assembly of pre-mRNA splicing machinery	[48,49]
	RIO kinase 1 (RioK1)	Recruits PRMT5 to nucleolin for methylation	Ribosomal synthesis and maturation	[50]

**Table 3:** Novel PRMT5 therapeutics and their current stages of development.

Company	Compound Name	Mode of Inhibition	Clinical Trial Stage	Trial Duration	Country	Types of Indications	References
Epizyme (sponsored by GSK)	EPZ015938/GSK3326595 (Pemrametostat)	Direct	Phase I/II	Phase I: 08/30/2016–04/29/2025 Phase II (03/21/2021–12/31/2022)	USA	Phase I: Solid tumors and non-Hodgkin’s lymphoma (with drug: pembrolizumab) Phase II: Early-stage breast cancer	(NCT02783300/NCT04676516) [87]
Johnson & Johnson	JNJ-64619178 (Onametostat)	Direct	Phase I	07/13/2018–12/30/2022	USA	Solid tumor, adult; non-Hodgkin lymphoma; myelodysplastic syndromes	NCT03573310 [89]
Pfizer	PF-06939999	Direct	Phase I	03/14/2019–04/14/2026	USA	Advanced or metastatic non-small cell lung cancer, head and neck squamous cell carcinoma, esophageal cancer, endometrial cancer, cervical cancer, bladder cancer (monotherapy, in combination with docetaxel)	NCT03854227 [90]
Prelude	PRT811	Direct	Phase I	11/06/2019–10/2022	USA	Advanced solid tumors, CNS lymphoma, and recurrent high-grade gliomas	NCT04089449
Prelude	PRT543	Direct	Phase I	02/11/2019–08/11/2022	USA	Relapsed/refractory advanced solid tumors; relapsed/refractory diffuse large B-cell lymphoma; relapsed/refractory myelodysplasia; relapsed/refractory myelofibrosis; adenoid cystic carcinoma; relapsed/refractory mantle cell lymphoma; relapsed/refractory acute myeloid leukemia; refractory chronic myelomonocytic leukemia	NCT03886831 [95]
EQon Pharmaceuticals	PR5-LL-CM01	Direct	Preclinical		USA	Pancreatic cancer/colorectal cancer (breast cancer)	[17]
GSK(license with Epizyme)	GSK3186000A	Direct	Preclinical		USA	Leukemia	[89]
Eli Lilly	LLY-283	Direct	Preclinical		USA	Skin cancer	[91]
Merck	CMP-5	Direct	Preclinical		USA	Glioblastoma	[93]
Miratis	MRTX9768	Targets PRMT5-MTA complex	Preclinical		USA	MTAPdel cancer cells	[94]

## Data Availability

Not applicable.
